# Modeling Human Hypertrophic Scars with Induced Pluripotent Stem-Cell-Derived Scar Organoids Versus Skin Organoids

**DOI:** 10.3390/cells15110969

**Published:** 2026-05-24

**Authors:** Hyun Mi Kim, Eun Jung Oh, Suin Kwak, Se Ok Han, Ho Yun Chung

**Affiliations:** 1 BK21 FOUR KNU Convergence Educational Program of Biomedical Science for Creative Further Talents, Department of Medical Science, Kyungpook National University, Daegu 41944, Republic of Korea; sarang7939@knu.ac.kr; 2 Department of Plastic and Reconstructive Surgery, Cell and Matrix Research Institute, School of Medicine, Kyungpook National University, Daegu 41944, Republic of Korea; fullrest74@knu.ac.kr (E.J.O.); suin8349@knu.ac.kr (S.K.); tpdhrgks@knu.ac.kr (S.O.H.)

**Keywords:** skin, organoids, hypertrophic scars, scar organoids, disease model, pluripotent stem cell, hiPSC, extracellular matrix

## Abstract

**Highlights:**

**What are the main findings?**

- An iPSC-derived scar organoid was established using TGF-β1 and hypoxic culture conditions.
- SCOs reproduced scar-associated features, including collagen accumulation, tissue contraction, and transcriptomic remodeling.

**What are the implications of the main findings?**

- SCOs may be useful for studying pathological skin remodeling and fibrosis in a human 3D platform.
- This model may support future mechanistic and translational studies related to hypertrophic scars.

**Abstract:**

Hypertrophic scars are characterized by excessive collagen deposition, fibrotic remodeling, and functional impairment. However, the ability of current models is limited in recapitulating human pathology. This study presents a novel approach using induced pluripotent stem cell-derived scar organoids to model hypertrophic scar characteristics in vitro. Following established protocols, human pluripotent stem cells were differentiated into skin organoids and induced fibrotic transformation by treatment with TGF-β1 (10 ng/mL) and hypoxia (5% O_2_) from day 45 onward. Scar organoids exhibited significant contraction and increased collagen I deposition compared with skin organoids. Immunofluorescence analysis showed reduced LHX2 expression, indicating loss of hair follicle development, while collagen I expression was significantly elevated. Dark-field imaging revealed marked morphological divergence between skin and scar organoids. RNA sequencing revealed distinct transcriptomic profiles. Expression of hair follicle-associated gene families (KRT and KRTAP) was upregulated in scar organoids, whereas epidermal structure-related genes (*KRT4*, *KRT7*, *CLDN7*, and *WNT7*) were downregulated. These findings demonstrate that iPSC-derived scar organoids successfully recapitulate key features of human hypertrophic scars, including excessive collagen production, loss of skin appendage development, and contractile behavior. This platform offers potential for future applications in drug screening, precision medicine, and understanding the molecular mechanisms underlying scar formation.

## 1. Introduction

The skin serves as a barrier organ that protects the body from the external environment and, following injury, restores structural and functional homeostasis through a finely regulated wound-healing process. Normal wound healing proceeds through sequential phases, including inflammation, proliferation, and remodeling, where coordinated interactions among keratinocytes, fibroblasts, endothelial cells, and immune cells are essential. However, disruption of these regulatory mechanisms can lead to excessive fibrotic responses, resulting in pathological scar formation [1,2,3,4,5,6,7].

The development of a hypertrophic scar (HS) is a pathological fibrotic response to skin injury characterized by excessive collagen production, extracellular matrix (ECM) accumulation, persistent fibroblast activation, and aberrant tissue remodeling. Meanwhile, normal wound healing is strictly regulated to restore tissue homeostasis through controlled inflammatory, proliferative, and remodeling phases. HS arises from dysregulated repair processes following deep dermal injury from trauma, surgery, or burns, leading to sustained tissue overgrowth and collagen deposition [8,9,10]. Clinically, HS presents as raised, erythematous lesions, often accompanied by pruritus, and remains confined within the original wound boundaries, in contrast to keloids, which extend beyond the initial injury site. These scars impose substantial functional, psychological, and socioeconomic burdens on patients, frequently causing pain, itching, joint contractures, and exhibiting high recurrence rates despite therapeutic intervention [11,12,13].

At the molecular and cellular levels, HS pathogenesis is driven by chronic inflammation, mechanical tension, and aberrant activation of profibrotic signaling pathways. In this pathological context, mechanotransduction induced by mechanical tension interacts with the transforming growth factor-β (TGF-β)/SMAD signaling pathway, thereby promoting sustained myofibroblast activation and persistent ECM accumulation [14,15,16,17]. Specifically, prolonged overactivation of TGF-β signaling facilitates fibroblast-to-myofibroblast differentiation and induces excessive production of collagenous ECM components, including collagen type I and collagen type III (COL1A1 and COL3A1), ultimately leading to progressive scar hypertrophy [18,19,20].

Histologically, HS tissue is characterized by thickened, irregularly arranged collagen bundles within the dermis and the prolonged presence of α-smooth muscle actin (α-SMA)-positive myofibroblasts, reflecting disruption of normal epidermal–dermal interactions [21,22]. Additionally, the loss of skin appendages, such as hair follicles and sebaceous glands, indicates impaired tissue regeneration. These structural alterations extend beyond cosmetic concerns, contributing to functional impairments including joint contractures, pain, and pruritus, and are associated with a high risk of recurrence, thereby representing a significant clinical burden [23,24].

Despite advances in understanding fibrotic signaling pathways, effective therapeutic options for HS remain limited, underscoring the need for physiologically relevant in vitro models that faithfully recapitulate the pathological features of human scarring [25,26].

Traditional approaches to HS research have primarily relied on two-dimensional (2D) fibroblast monolayer cultures and animal models. While 2D cultures are useful for mechanistic studies and high-throughput screening, these cultures fail to reproduce the three-dimensional (3D) tissue architecture, mechanical cues, cell–cell interactions, and tissue complexity that critically influence fibroblast behavior and ECM organization in vivo. Consequently, such reductionist systems do not adequately model the complex, multicellular, 3D nature of human HSs [27,28,29].

Animal models, particularly genetically engineered mouse models, have provided valuable insights into wound healing and fibrosis; however, interspecies differences in skin thickness, hair density, immune responses, and fibrotic tendencies limit the ability of these models to mimic human hypertrophic scarring fully. Moreover, variability in genetic background and experimental conditions can compromise phenotypic consistency, increasing the time and cost required for translational research [30,31].

Indeed, 3D spheroid models have also been introduced to study fibrosis; however, these systems typically consist of a single cell type and do not sufficiently recapitulate the layered architecture and appendage development unique to human skin. These limitations further highlight the need for human-specific models capable of bridging the gap between basic research and clinical application [32].

Recent advances in induced pluripotent stem cell (iPSC) technology have provided a powerful platform for generating human-specific disease models. Notably, iPSCs are pluripotent stem cells generated by reprogramming somatic cells with Yamanaka factors and possess self-renewal capacity and the ability to differentiate into diverse skin-related cell lineages, while avoiding the ethical concerns associated with human embryonic stem cells [33,34,35,36,37]. Furthermore, iPSCs are amenable to genetic manipulation, making these cells valuable tools for the development and validation of gene-based therapeutic strategies. Building on this technology, iPSC-derived 3D organoids enable the generation of complex tissue-like structures that recapitulate key aspects of human organ development and function [38,39].

In particular, iPSC-derived skin organoids (SKOs) self-organize into multilayered epidermal and dermal structures and form skin appendages, including hair follicles, sebaceous glands, Merkel cells, and eccrine sweat glands, thereby closely recapitulating human fetal skin architecture [40]. Thus, owing to this structural complexity and cellular diversity, SKOs have emerged as promising platforms for studying human skin diseases that are difficult to model using conventional systems. However, the application of iPSC-derived organoids to pathological scar modeling remains limited [41,42,43].

Therefore, this study aimed to establish a novel in vitro model that physiologically recapitulates the pathological features of human HS using iPSC-derived organoid technology. Indeed, by inducing hypertrophic scar organoids (SCOs) based on established protocols for human skin organoid generation and directly comparing this lineage with normal SKOs, this study sought to characterize the structural and molecular features of HS within a 3D context. This study proposes iPSC-derived SCOs as a human-specific 3D platform for investigating HS pathogenesis and evaluating therapeutic candidates in a biologically relevant setting.

## 2. Materials and Methods

### 2.1. Generation and Culture of Human iPSCs

The study was performed in accordance with the Declaration of Helsinki and approved by the Institutional Review Board of Kyungpook National University Hospital (Approval No. KNUH 2023-04-004-002; approved on 9 June 2023). Informed consent was obtained from the donor. Human dermal fibroblasts were isolated from tissue samples, and the human iPSCs used in this study were generated from dermal fibroblasts obtained from single donor. Briefly, tissues were treated with dispase overnight to isolate the dermal layer, followed by removal of subcutaneous fat. The dermal tissue was digested with collagenase type II for 1 h and passed through a 70 μm cell strainer (Corning®, Corning, NY, USA). The resulting cells were collected and maintained in Dulbecco’s modified Eagle medium (DMEM) HIGH (Hyclone, Logan, UT, USA) under standard culture conditions.

Human induced pluripotent stem cells (hiPSCs) were generated from dermal fibroblasts using a non-modified RNA (NM-RNA)-based reprogramming system under completely xeno-free conditions. Reprogramming was performed using the StemRNA^TM^ 3rd Gen Reprogramming Kit (Cat. No. 00-0076, Stemgent^®^, Yokohama, Japan) according to the manufacturer’s instructions. Fibroblasts at approximately 80% confluence were used for reprogramming. Before NM-RNA transfection, the culture medium was replaced with NutriStem Medium for 30 min. Subsequently, 500 μL of the NM-RNA reprogramming transfection mixture was added to the fibroblasts, followed by overnight incubation at 37 °C in a humidified 5% CO_2_ atmosphere. This transfection procedure was repeated every 24 h for four consecutive days. From day 5 onward, the medium was replaced daily with fresh NutriStem Medium.

During reprogramming, fibroblasts gradually underwent morphological changes, becoming shorter and forming compact colonies. Between days 10 and 14, colonies with diameters of approximately 500 μm or greater were manually selected and transferred onto vitronectin-coated dishes (VTN-N, A31804, Gibco, NY, USA) containing TeSR^TM^-E8^TM^ medium (05990, STEMCELL Technologies, Vancouver, BC, Canada).

The hiPSCs were maintained at 37 °C in a humidified 5% CO_2_ atmosphere and passaged every 5 days using UltraPure^TM^ 0.5 M EDTA (pH 8.0; 15575-020, Gibco, USA) in the presence of the ROCK inhibitor Y-27632 (1254, Tocris, Bristol, UK). All hiPSCs used in this study were maintained within 10 passages.

### 2.2. Generation and Culture of iPSC-Derived SKOs and SCOs

SKOs were generated from hiPSCs using a previously established protocol with modifications [44]. Briefly, hiPSCs were aggregated to form embryoid bodies, which were then sequentially differentiated into surface ectoderm and mesenchymal lineages. This was followed by self-organization into 3D SKOs. From day 0 to day 45, the organoids were generated according to the published protocol, which resulted in the formation of stratified epidermal layers and dermal-like compartments.

After 45 days, the organoids were maintained in organoid maturation medium (OMM) under orbital shaking conditions (65 rpm) to promote further maturation and tissue organization. These organoids were defined as SKOs and cultured long-term for further maturation.

**Figure 1:**
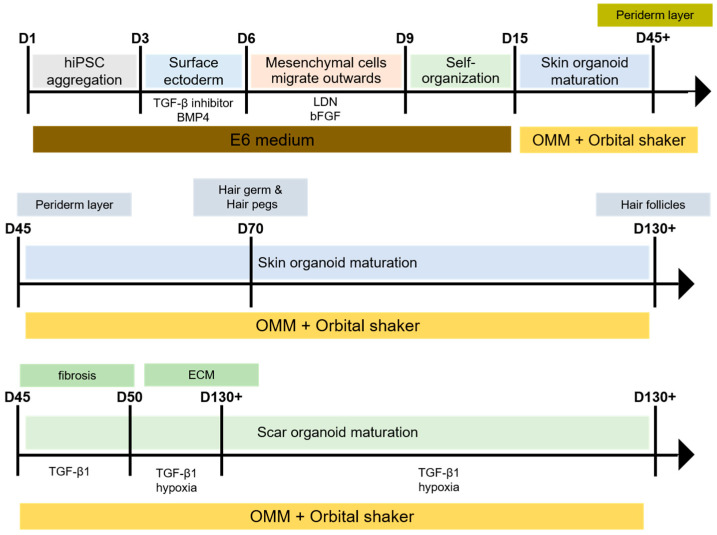
Overview of the generation and maturation of iPSC-derived SKOs and SCOs. Briefly, hiPSCs were aggregated and sequentially differentiated into SKOs using a stepwise protocol that involved surface ectoderm induction, mesenchymal specification, and self-organization. During early differentiation (days 1–6), hiPSC aggregates were cultured in E6 medium supplemented with a TGF-β inhibitor, BMP4, LDN, and bFGF to induce surface ectoderm formation. From days 6 to 12, mesenchymal cells migrated outward, followed by self-organization and commitment to the skin lineage. SKOs were subsequently matured in organoid maturation medium (OMM) under orbital shaking, resulting in the formation of periderm layers (day 45+), hair germ and hair peg structures (day 70), and mature hair follicles (day 130+). For the generation of SCOs, SKOs on day 45 were exposed to fibrotic stimuli, including TGF-β1 and hypoxia, to induce fibrosis and ECM remodeling. SCOs were further matured under continuous OMM culture with orbital shaking until day 130+, resulting in stable fibrotic organoid structures that recapitulate key pathological features of HS.

To generate SCOs, pre-established SKOs at day 45 were subjected to fibrosis-inducing conditions. SKOs were cultured in OMM supplemented with TGF-β1 (10 ng/mL) under hypoxic conditions (5% O_2_). Fibrotic induction was maintained during subsequent culture to promote ECM remodeling and scar-like tissue formation. These fibrotically induced organoids were defined as SCOs and maintained for downstream analyses (Figure 1). Technical replicates represent independently cultured organoids generated from a single donor-derived hiPSC line.

### 2.3. Morphological and Quantitative Analysis of Organoids

Bright-field (BF) imaging was performed using an inverted microscope (Axio, Carl Zeiss, Jena, Germany) equipped with an objective lens to monitor organoid development up to 90 days of differentiation. Dark-field images were obtained using an upright microscope system (Ni-E, Nikon, Tokyo, Japan) on days 110, 130, and 155 to visualize mature organoids.

For quantitative analysis, SKOs and SCOs were pooled at each time point, and BF images were captured for size measurement. The diameter of individual organoids was quantified from the acquired images using Fiji ImageJ software version 1.54h (National Institutes of Health, Bethesda, MD, USA). The measured diameters were plotted as bar graphs using GraphPad Prism version 10.5.0 (GraphPad Software, San Diego, CA, USA).

### 2.4. Histological and Immunofluorescence Analyses

Organoid samples were fixed in 10% paraformaldehyde (PFA), embedded in paraffin, and sectioned to a thickness of 10 μm. Histological staining was performed using H&E and Masson’s trichrome, following standard protocols.

For immunofluorescence analysis, paraffin sections were deparaffinized and rehydrated, followed by antigen retrieval using a citrate acid buffer. The sections were then permeabilized and incubated overnight (≥16 h) at 4 °C with primary antibodies against collagen I (COL1; ab138492, Abcam, Cambridge, UK) and LHX2 (SC-517243, Santa Cruz Biotechnology, Dallas, TX, USA). After washing, the sections were incubated with secondary antibodies, goat anti-rabbit IgG (H+L) Alexa Fluor® 594 (A-11012, Invitrogen, Waltham, MA, USA) and goat anti-mouse IgG (H+L) Alexa Fluor® 594 (A-11001, Invitrogen, Waltham, MA, USA), at room temperature in the dark for 3 h. Nuclei were counterstained with DAPI (hydrochloride; 75004, STEMCELL Technologies, Vancouver, BC, Canada) for 10 min. The sections were mounted using VECTASHIELD® Antifade Medium (H-1000, Vector Laboratories, Newark, CA, USA). Immunofluorescence images were acquired using a Leica DMi8 THUNDER fluorescence microscope (Leica Microsystems, Wetzlar, Germany).

### 2.5. RNA Sequencing and Transcriptomic Analyses

Total RNA was extracted from organoid samples using TRIzol reagent (15596026, Invitrogen™), and RNA quality was assessed before library preparation. RNA sequencing libraries were prepared and sequenced using standard protocols. Briefly, full-length cDNA was generated using an LNA-containing template-switching oligonucleotide-based method, followed by Tn5 transposase-mediated adapter tagmentation and selective amplification. Libraries were sequenced on an Illumina NovaSeq X Plus platform with paired-end 151 bp reads. Raw reads were quality-filtered and aligned to the human reference genome (GRCh38) using a splice-aware aligner. PCR-derived duplicate reads were removed, and uniquely mapped reads were used for gene-level quantification based on genome annotation files. Differential gene expression analysis was performed using DESeq2 with default parameters. Adjusted *p*-values were calculated using Benjamini–Hochberg false discovery rate (FDR) correction method. Differentially expressed genes were identified using thresholds of |log2 fold-change | ≥ 2 and adjusted *p*-value ≤ 0.01. For visualization and multivariate analyses, including heatmaps and principal component analysis, gene expression data were normalized using rlog transformation. The full list of differentially expressed genes is provided in Supplementary Table S1.

### 2.6. Statistical Analysis

Data are presented as the mean ± standard deviation (SD) from three technical replicates. Group differences were analyzed using one-way analysis of variance (ANOVA) followed by Dunnett’s post hoc test for multiple comparisons against the control. Statistical significance was defined as a *p*-value ≤ 0.05. All statistical analyses were conducted using GraphPad Prism version 10.5.0 (GraphPad Software, San Diego, CA, USA).

## 3. Results

### 3.1. Generation and Morphological Development of iPSC-Derived SKOs and SCOs

#### Morphological Development of SKOs and SCOs

The following representative bright-field (BF) images illustrate the temporal progression of organoids: (a) SKO development from hiPSC aggregation (day 0) through the early differentiation and self-organization stages (days 3–38) to organoid formation (day 45). During differentiation, progressive morphological changes were observed, including organoid compaction, cavity formation, and tissue thickening. (b) After 45 days, the pre-established organoids were cultured under either standard maturation conditions to maintain SKO identity or fibrotic induction conditions to generate SCOs. Representative images of SKOs and SCOs at days 47, 56, and 90 demonstrate the divergent morphological features that arise after lineage bifurcation. SCOs exhibit increased tissue density and altered structural organization compared with SKOs. Scale bar, 100 μm.

**Figure 2:**
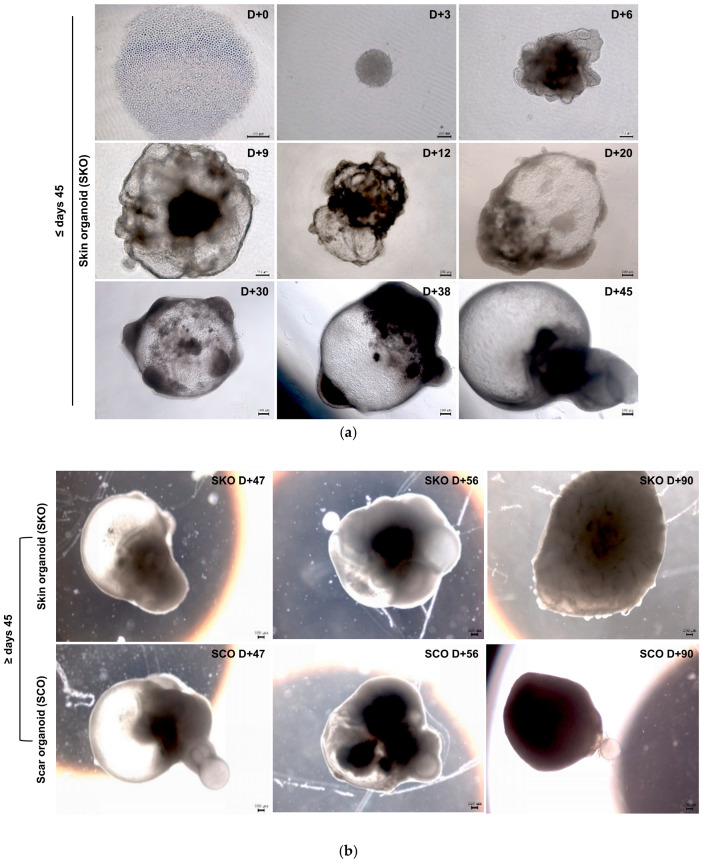
Development of induced pluripotent stem cell (iPSC)-derived skin organoids (SKOs) and subsequent generation of scar organoids (SCOs). (**a**) Schematic overview of SKO development during early differentiation stages before 45 days. (**b**) Representative images of SKOs and SCOs after 45 days under standard maturation or fibrotic induction conditions. Scale bar, 100 μm.

To establish a human in vitro model of skin and scar tissue, we monitored the gradual development of hiPSC-derived SKOs. Following hiPSC aggregation, the organoids underwent progressive morphological changes, including compaction and self-organization during differentiation. Ultimately, well-defined SKOs had formed by day 45. These temporal morphological transitions reflect the ordered progression of skin lineage specification and tissue organization (Figure 2a).

After 45 days, the pre-established organoids were cultured under different conditions to generate either mature SKOs or SCOs. Notably, SKOs maintained relatively stable and organized morphologies during prolonged maturation, whereas SCOs derived from SKOs displayed significant structural alterations over time. In particular, SCOs exhibited increased tissue density and a more compact morphology indicative of fibrotic remodeling. These observations demonstrate that SCOs can be consistently generated from pre-existing SKOs by modulating culture conditions after differentiation (Figure 2b).

### 3.2. Quantitative Analysis of Organoid Growth Dynamics

#### Diameter-Based Comparison of SKOs and SCOs at Defined Time Points

To quantitatively compare the growth characteristics of SKOs and SCOs, diameters were measured on day 45, which represents the stage before divergence, and on days 90, 110, and 130. Following lineage bifurcation after day 45, SKOs exhibited a progressive increase in diameter over time. In contrast, SCOs showed a comparatively restricted growth pattern. By day 90, SCOs had a significantly smaller diameter than SKOs. These differences became more pronounced at later stages, with SCOs on days 110 and 130 exhibiting markedly reduced diameters relative to SKOs at the corresponding time points.

**Figure 3:**
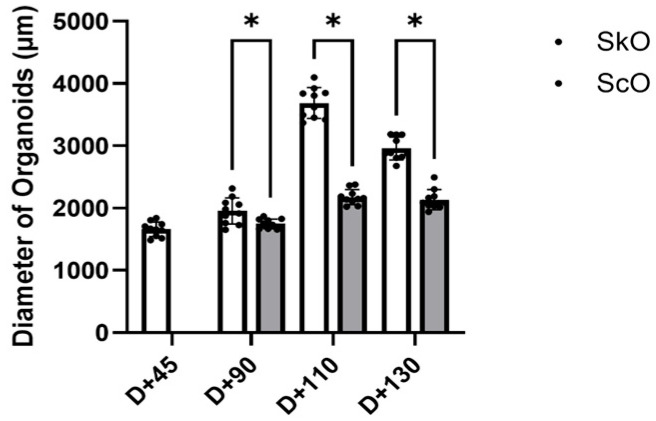
Quantitative comparison of the diameters of SKOs and SCOs at defined maturation stages. The diameters of SKOs and SCOs were measured on day 45, before divergence, and on days 90, 110, and 130 using BF microscopy and ImageJ software. Data were analyzed using one-way analysis of variance (ANOVA) with Dunnett’s post hoc test. Data are presented as mean ± standard deviation (SD) (n = 10 organoids per group); * p ≤ 0.05.

These findings reveal divergent growth patterns between SKOs and SCOs following lineage bifurcation (Figure 3).

### 3.3. Histological Characterization of SKOs and SCOs

#### Tissue Architecture and ECM Remodeling in SKOs and SCOs

**Figure 4:**
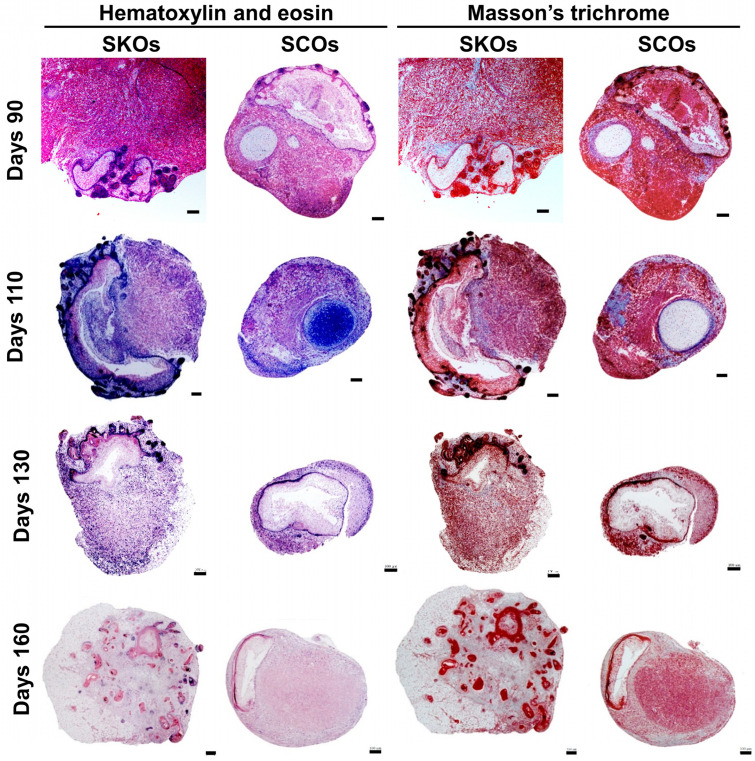
Representative hematoxylin and eosin (H&E) and Masson’s trichrome-stained sections of SKOs and SCOs at days 90, 110, 130, and 160 of differentiation. H&E staining illustrates overall tissue architecture and structural organization. Masson’s trichrome staining highlights ECM and collagen-rich regions. Compared with SKOs, SCOs exhibit increased tissue compactness and enhanced collagen deposition over time, indicating progressive remodeling of tissue structure during prolonged culture. (n = 3 organoid per group). Scale bar, 100 μm.

Histological analyses were performed to evaluate tissue architecture and ECM organization in SKOs and SCOs at maturation stages corresponding to days 90, 110, 130, and 160. At day 90, hematoxylin and eosin (H&E) staining revealed an organized tissue structure in SKOs, whereas SCOs exhibited increased tissue compactness and altered architectural features. Masson’s trichrome staining showed increased collagen-rich matrix deposition in SCOs compared with SKOs, indicating early ECM remodeling. These histological differences became more pronounced over time. By days 110 and 130, SCOs displayed denser tissue organization and expanded regions of collagen accumulation, while SKOs maintained more defined epithelial and stromal compartmentalization. By day 160, SCOs exhibited extensive ECM deposition and markedly compact tissue morphology. In contrast, SKOs preserved relatively stable structural features. Collectively, histological evaluation revealed a time-dependent divergence in tissue organization between SKOs and SCOs, with SCOs showing progressive collagen enrichment and structural remodeling during prolonged culture (Figure 4).

### 3.4. Immunofluorescence Analysis of Fibrotic and Skin-Associated Markers

#### Differential Expression of COL1 and LHX2 in SKOs and SCOs

**Figure 5:**
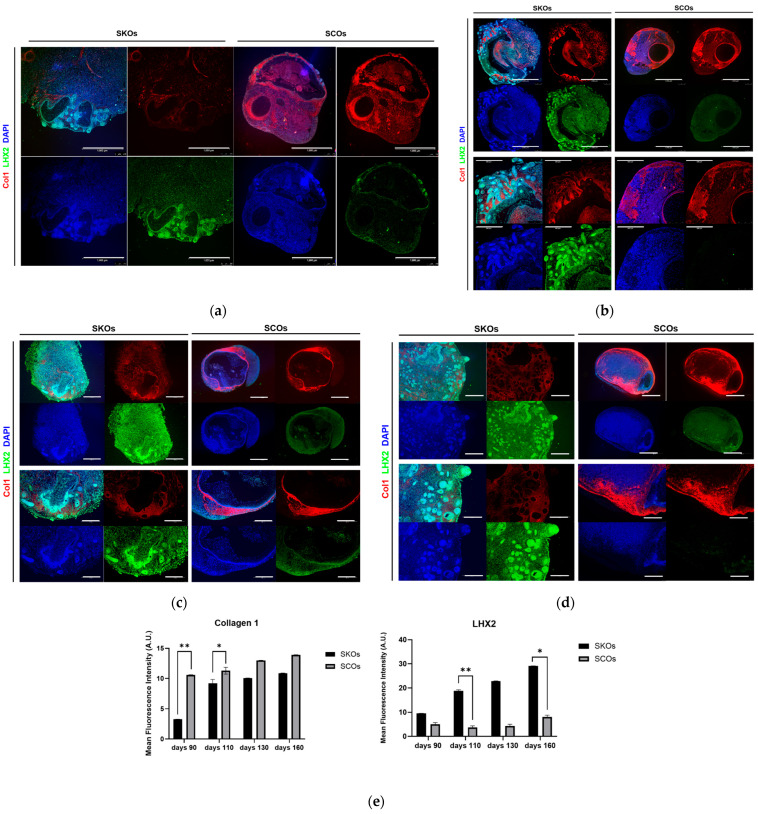
Immunofluorescence analysis of COL1 and LHX2 expression in SKOs and SCOs during long-term maturation. Representative immunofluorescence images of the expression on dermal layer markers (collagen I, COL1) and hair follicle-associated markers (LIM homeobox 2, LHX2) expression in SKOs and SCOs at (**a**) day 90, (**b**) day 110, (**c**) day 130, and (**d**) day 160 of differentiation. Nuclei were counterstained with DAPI. (**e**) Quantification of the mean fluorescence intensity (MFI) of COL1 and LHX2 in SKOs and SCOs at the indicated time points. Data are presented as mean ± SD (n = 3 organoid per group); * p ≤ 0.05, ** p ≤ 0.01. Scale bars: (**a**,**b**) 1000 μm; (**c**,**d**) 200 μm.

Immunofluorescence analysis was performed to evaluate COL1 and LHX2 expression patterns in SKOs and SCOs at the following maturation stages: days 90, 110, 130, and 160. At day 90, SKOs exhibited distinct LHX2-positive regions, whereas SCOs showed more prominent COL1 signals (Figure 5a). These differences became more evident at later time points. At days 110 and 130, SCOs showed increased COL1 fluorescence intensity compared with SKOs, while LHX2 expression was reduced and spatially altered in SCOs (Figure 5b,c). By day 160, SCOs exhibited strong and widespread COL1 signals, whereas SKOs maintained comparatively lower COL1 expression and preserved LHX2-positive regions (Figure 5d). Quantitative analysis of mean fluorescence intensity further supported these observations, demonstrating higher COL1 levels and lower LHX2 levels in SCOs than in SKOs across all examined time points (Figure 5e). Collectively, these findings reveal distinct temporal expression patterns of COL1 and LHX2 between SKOs and SCOs during prolonged culture.

### 3.5. Morphological Characteristics of SKOs and SCOs

#### Dark-Field Imaging of SKOs and SCOs During Late-Stage Maturation

**Figure 6:**
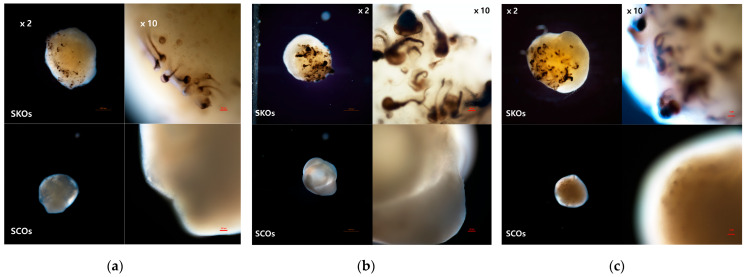
Representative dark-field SKOs (upper) at (**a**) day 110, (**b**) day 130, and (**c**) day 160 are shown at low (×2) and high (×10) magnification. SKOs exhibit prominent pigmented structures and complex surface protrusions consistent with hair-follicle-like appendage formation, whereas SCOs display a smooth, compact morphology with reduced surface complexity across all time points. Scale bar, 100 μm.

Dark-field imaging revealed distinct morphological differences between SKOs and SCOs during late-stage maturation. At days 110, 130, and 160, SKOs consistently exhibited a heterogeneous surface morphology with multiple pigmented, protrusive structures, indicative of skin appendage-associated differentiation. These features became more pronounced with prolonged culture, reflecting the continued structural maturation of SKOs. In contrast, SCOs displayed a markedly different morphological profile. At all examined time points, SCOs maintained a smooth, compact, and relatively featureless surface, lacking the pigmented protrusions observed in SKOs. Overall, SCOs appeared more condensed and uniform, suggesting suppression of normal skin appendage development and altered tissue organization. Dark-field imaging further showed that SCOs underwent persistent morphological remodeling, characterized by a loss of surface complexity and appendage-like structures, supporting the divergence of these organoids from SKOs during late-stage maturation (Figure 6).

### 3.6. Transcriptomic Profiling of SKOs and SCOs

#### Differential Gene Expression and Fibrosis-Associated Pathways in SKOs and SCOs

**Figure 7:**
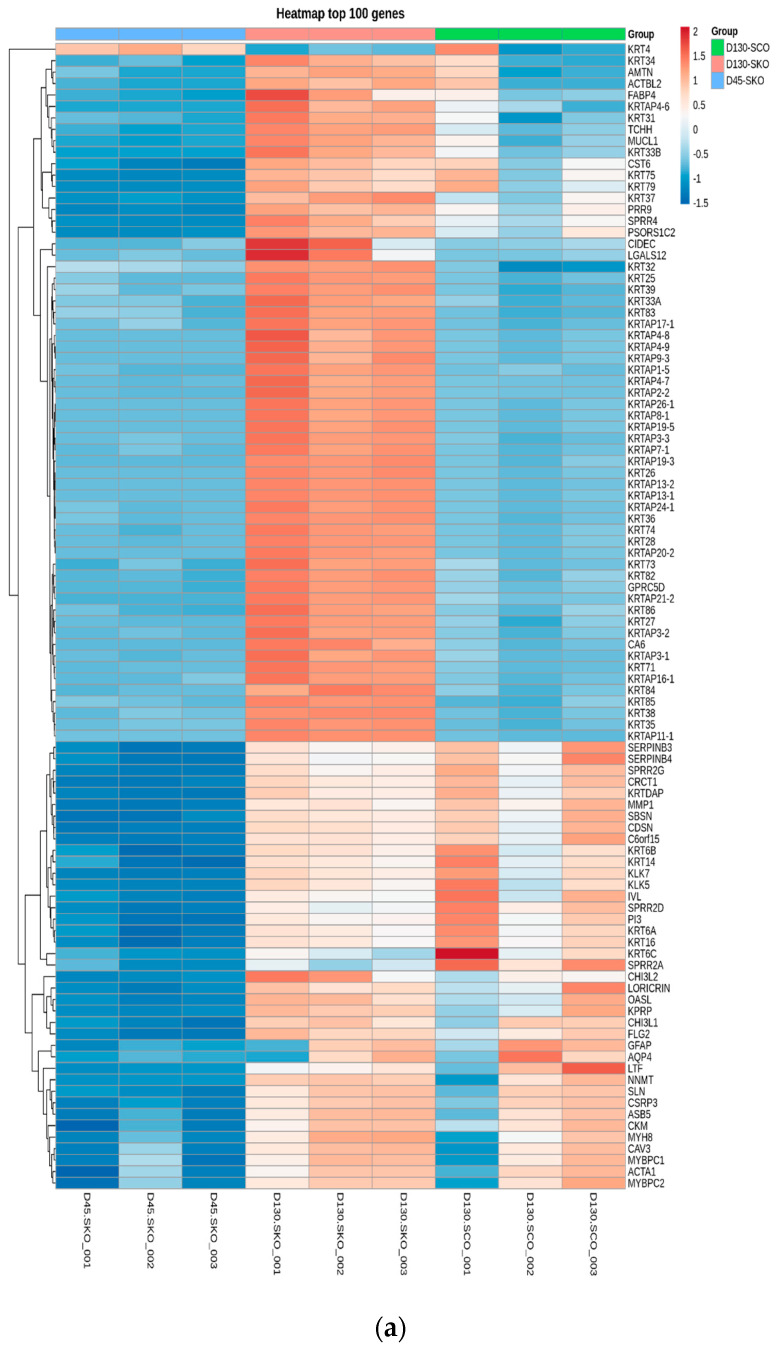

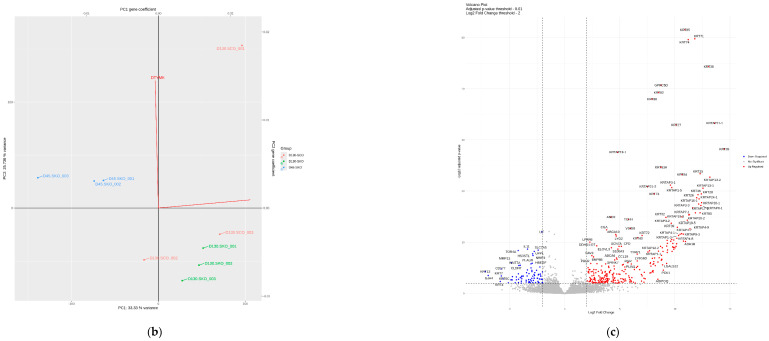

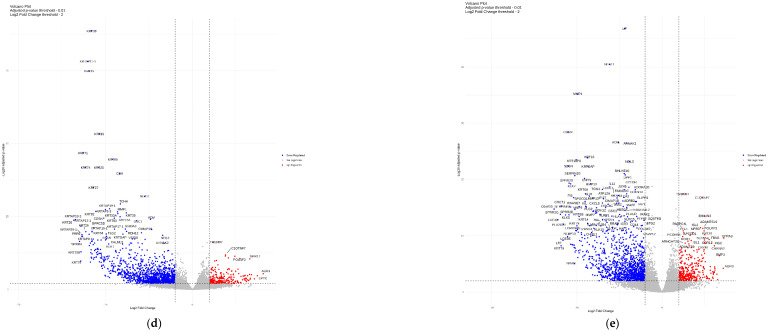
Transcriptomic profiling of SKOs and SCOs at defined maturation stages. (**a**) Heat map of differentially expressed genes (DEGs) showing hierarchical clustering of day 130 SCOs (D130-SCOs, green), day 130 SKOs (D130-SKOs, red), and day 45 SKOs (D45-SKOs, blue). Gene expression values were log-ratio (rlog)-transformed and Z-score-normalized for visualization. (**b**) Principal component analysis (PCA) demonstrates clear separation of transcriptomic profiles among day 45 SKOs, day 130 SKOs, and day 130 SCOs based on rlog-transformed gene expression data. (**c**) Volcano plot of the differentially expressed genes (DEGs) between day 130 SKOs and day 130 SCOs (276 upregulated and 80 downregulated). (**d**) Volcano plot of the DEGs between days 45 SKOs and days 130 SKOs (255 upregulated and 1253 downregulated). (**e**) Volcano plot of the DEGs between day 45 SKOs and days 130 SCOs (339 upregulated and 1212 downregulated). Genes were classified as significantly upregulated or downregulated based on a |log2 fold-change |≥ 2 and adjusted *p*-value ≤ 0.01. Representative genes with large fold changes and high statistical significance are labeled.

To investigate transcriptomic changes associated with SKO and SCO maturation, RNA sequencing was performed on day 45 SKOs (D45-SKOs), day 130 SKOs (D130-SKOs), and day 130 SCOs (D130-SCOs). Hierarchical clustering of differentially expressed genes (DEGs) revealed distinct transcriptional profiles among the three groups (Figure 7a). In particular, day 130 SCOs formed a separate cluster from both day 130 SKOs and day 45 SKOs, indicating substantial transcriptomic divergence following SCO induction. Principal component analysis further demonstrated clear segregation of samples according to organoid type and maturation stage (Figure 7b). Differential gene expression analysis using volcano plots demonstrated marked transcriptomic differences between the experimental groups. Specifically, the volcano plot analysis revealed a pronounced and significant transcriptional difference between day 130 SKOs and day 130 SCOs. Moreover, the analysis identified 276 upregulated genes and 80 downregulated genes, reflecting changes in gene expression associated with SCO formation. Notably, multiple hair follicle-associated keratin (KRT) genes and KRT-associated protein (KRTAP) genes were strongly upregulated in day 130 SCOs, including members of the KRT and KRTAP gene families. In contrast, genes associated with epidermal structure and cell junction organization, such as KRT4, KRT7, CLDN7, and WNT7A, were downregulated in day 130 SCOs relative to day 130 SKOs (Figure 7c). In comparison between day 45 SKOs and day 130 SKOs, a large number of genes were significantly downregulated in day 130 SKOs, particularly those associated with keratinocyte differentiation and epidermal structural organization. Representative downregulated genes included multiple members of the KRT and KRTAP families, as well as SPRR genes. In contrast, a smaller subset of genes was upregulated in day 130 SKOs, including AGR3, EPYC, POU4F3, GPR17, and C1QTNF7. These transcriptional changes indicate extensive remodeling of gene expression programs during prolonged SKO maturation. Collectively, the volcano plot analysis demonstrates that SKOs undergo marked transcriptomic reorganization between day 45 and day 130, characterized by a reduced expression profile associated with later-stage maturation (Figure 7d). Distinct gene expression patterns were also observed between day 45 SKOs and day 130 SCOs. Genes associated with keratinocyte differentiation and epidermal structural organization were predominantly reduced in day 130 SCOs, including members of the KRT and SPRR gene families, as well as loricrin and LCE. Conversely, day 130 SCOs showed increased expression of POU4F3, AGR3, GPR17, C1QTNF7, BMP3, and ADAMTS19, reflecting the emergence of gene expression programs distinct from those observed in early-stage SKOs (Figure 7e). These results indicate that SCOs exhibit a markedly divergent transcriptional signature from early-stage SKOs, characterized by the suppression of epidermal differentiation-associated genes and the induction of alternative gene expression programs associated with late-stage SCO maturation.

## 4. Discussion

By modifying the established SKO protocol with TGF-β1 supplementation and hypoxic culture conditions, the organoids exhibited scar-like characteristics, including increased collagen deposition, loss of hair follicle structures, and tissue contraction.

We chose to build upon the published iPSC-derived SKO protocol, which successfully generates hair-bearing skin structures with stratified epidermis and appendages [44,45]. This provided an ideal baseline for modeling the transition from normal wound healing to pathological scarring. TGF-β1 was selected as the primary profibrotic stimulus based on the associated well-established role as a master regulator of fibrosis across multiple organ systems [46]. Indeed, TGF-β1 induces collagen expression and accumulation via the canonical Smad3 pathway, with Smad3 phosphorylation at Ser423/425 serving as a critical activation step [47,48,49]. In the context of scar formation, TGF-β1 promotes fibroblast-to-myofibroblast transition (FMT), characterized by expression of α-SMA and excessive synthesis of ECM components, including collagen type I and collagen type III [50]. Studies using vascularized skin equivalents have demonstrated that TGF-β exposure recapitulates key features of skin, including activation of TGF-β pathways, myofibroblast transition, and excessive ECM deposition [51]. The concentration of 10 ng/mL used in our study was based on established protocols for inducing fibrotic transformation in 3D tissue models [52,53,54,55]. Hypoxic culture conditions (5% O_2_) were incorporated based on compelling evidence linking tissue hypoxia to pathological scar formation [56,57,58]. Wounds known to produce HSs exhibit immediate relative hypoxia and increased expression of hypoxia-inducible factor-1α (HIF-1α) compared with normal wounds [59]. Mechanistically, hypoxia drives the transition of dermal fibroblasts to a myofibroblast-like phenotype via the TGF-β1/Smad3 pathway, and HIF-1α activates TGF-β1/Smad3 signaling to increase collagen deposition in dermal fibroblasts [60,61]. The synergistic interaction between TGF-β1 and hypoxia has been demonstrated previously, thereby supporting our rationale for combining these two stimuli to generate a robust scar model [62,63]. In addition, hypoxia-associated HIF-1α stabilization is known to cooperate with TGF-β signaling to promote fibroblast activation, ECM deposition, and fibrotic remodeling in pathological scars [62]. Therefore, the combined application of hypoxia and TGF-β1 in the present study may have synergistically contributed to the induction of scar-associated phenotypes in SCOs.

Meanwhile, H&E and Masson’s trichrome staining revealed several hallmark features of HSs in SCOs compared with SKOs. The increased dermal thickness, disorganized collagen architecture, and presence of collagen nodules observed in SCOs closely resemble human HSs [64,65]. These findings align with established histological criteria for identifying pathological scars, which include thickened epidermis and dermis and disorganized collagen fibers appearing in nodular formations. The collagen deposition patterns observed in SCOs are similar to those reported in animal models of hypertrophic scarring [66]. Similar features were observed in SCOs, including the absence of the organized, wavy collagen pattern typical of normal skin and the presence of dense, disorganized collagen fibers. These histological similarities support the relevance of SCOs in recapitulating key features of HSs [59].

The selection of COL1 and LHX2 as immunofluorescence markers was intended to assess fibrotic remodeling and regenerative capacity within the SCOs. COL1 was chosen as a representative marker of fibrosis because this is the most abundant collagen protein in HSs and a major product of myofibroblast activity [67]. Single-cell transcriptomic studies have identified fibroblast subpopulations in HSs characterized by high expression of COL1A1, COL3A1, and CTHRC1, which constitute the dominant cellular population. In addition, autocrine COL1A1/2–CD44 signaling has been reported to be upregulated in HS fibroblasts, further supporting COL1 as a key indicator of fibrotic activation [68]. Consistent with these findings, increased COL1 immunofluorescence in SCOs compared with SKOs reflects enhanced fibrotic remodeling in the scar model [13]. LHX2 was selected as a marker of hair follicle development and regenerative potential, providing a complementary perspective on the scar phenotype. LHX2 plays a critical role in hair follicle morphogenesis through the NF-κB/LHX2/TGF-β2 signaling axis and serves as a specific marker for hair follicle identification [69]. Notably, LHX2 expression during wound healing has been associated with regenerative responses, whereas LHX2 downregulation correlates with scar formation rather than tissue regeneration. The reduced LHX2 expression observed in SCOs indicates impaired hair follicle regeneration, a defining characteristic of pathological scarring in which appendage structures are typically absent [70]. Together, increased COL1 expression and decreased LHX2 expression provide molecular evidence that SCOs capture the shift from regenerative to fibrotic wound healing.

Tissue contraction is a functional feature of HSs. Therefore, this study quantitatively assessed tissue contraction by serially measuring organoid diameter. Scar contraction is primarily mediated by myofibroblasts, which generate contractile forces through α-SMA expression and contribute to abnormal tissue remodeling and functional impairment in pathological scars [71]. The progressive reduction in diameter observed in SCOs compared with SKOs indicates enhanced contractile activity within the scar model. Similar contractility has been reported in tissue-engineered keloid models, in which increased contraction is associated with myofibroblast-enriched fibroblast populations [72]. These findings suggest that SCOs recapitulate a functionally relevant contractile phenotype characteristic of scars. Mechanical tension is a key pathological feature of HS formation. Although the present SCO system was maintained under floating culture conditions without externally applied mechanical loading, the organoids nevertheless demonstrated contraction, ECM remodeling, and fibrosis-associated transcriptomic changes, suggesting intrinsic tissue remodeling dynamics within the 3D culture. However, the absence of controlled biomechanical stimulation remains an important limitation of the current model and may be addressed in future mechanically integrated platforms.

Bulk RNA sequencing revealed marked transcriptional differences between SKOs and SCOs throughout organoid maturation, including upregulation of ECM-related genes, activation of profibrotic pathways, and substantial alterations in epidermal differentiation programs. Hierarchical clustering and principal component analysis demonstrated that day 130 SCOs formed a distinct transcriptional cluster, separated from both day 45 SKOs and day 130 SKOs, indicating that fibrotic induction leads to a transcriptional state distinct from SKO maturation. Differential gene expression analysis further demonstrated pronounced transcriptomic divergence between day 130 SKOs and day 130 SCOs, with a large subset of genes differentially regulated following SCO induction. Recently transcriptomic and RNA sequencing studies of HS have identified molecular signatures associated with extracellular matrix remodeling, fibroproliferative activation, and inflammatory signaling. Consistent with these observations, transcriptomic analysis of SCOs revealed increased expression of fibrosis and remodeling of associated genes, including COL1A1, COL3A1, MMP2, LOX/LOX1, and CXCR12, as well as inflammatory chemokines such as CCL19 and CCL8 [73,74,75,76,77,78,79,80,81,82]. These findings suggest that SCOs partially recapitulate clinically relevant hypertrophic scar-associated molecular features. Notably, epidermal differentiation-associated genes were broadly downregulated in SCOs compared with SKOs, including multiple gene families associated with KRT, KRTAPs, and the cornified envelope, consistent with prior reports describing impaired epidermal differentiation and loss of hair-follicle-associated KRT programs in fibrotic skin and HSs. Interestingly, this finding supports the notion that SCOs recapitulate the molecular features of pathological skin remodeling rather than those of physiological maturation [83,84,85]. Although a similar attenuation of epidermal and keratinocyte differentiation markers was also observed during late-stage SKO maturation, the transcriptional profile of SCOs exhibited additional divergence beyond maturation-associated remodeling.

Although bulk RNA sequencing revealed upregulation of genes related to KRT and KRTAPs in day 130 SCOs, this finding does not indicate preserved hair follicle development. SCOs exhibited markedly reduced expression of LHX2, a regulator of hair follicle morphogenesis, together with markedly reduced follicular structures at the morphological level. Importantly, expression of the KRT and KRTAP gene families is not exclusively linked to functional hair follicle formation and can be aberrantly induced under fibrotic or wound-associated conditions [86,87,88,89]. Functional enrichment analysis further suggested enrichment of keratinization and epithelial remodeling-associated programs rather than preservation of mature follicular differentiation. Thus, the upregulation of selected KRT and KRTAP gene families in SCOs likely reflects a scar-associated epithelial state rather than bona fide hair follicle differentiation.

Collectively, these structural, functional, and molecular findings highlight the broader applicability of SCOs. The iPSC-derived SCOs address limitations of conventional 2D cultures and animal models by enabling analysis of human skin architecture and multicellular interactions in 3D. Additionally, the scalability and reproducibility of these models may support future mechanistic studies of scar formation and translational applications, including patient-specific modeling and the development of antifibrotic drug screening platforms. Future studies evaluating therapeutic responses to antifibrotic agents will also be important for further validating the translational applicability of the SCO model.

This study presents an initial iPSC-derived SCO model. However, several limitations should be acknowledged, including the absence of vascular and immune components, such as macrophage-driven inflammatory cues; the use of a single donor-derived hiPSC line, which may limit the assessment of inter-line variability; a prolonged culture duration of more than 90 days, which may constrain certain applications; the need for broader validation using multi-donor human scar tissues at both histological and transcriptomic levels, as well as longitudinal single-cell analyses, to further enhance the physiological relevance and translational utility of this model. In addition, the current SCO model was established using a single donor-derived hiPSC line under floating culture conditions without external mechanical loading. Although SCOs exhibited intrinsic tissue remodeling and contractile features, these limitations may affect the physiological complexity and translational applicability of the model. Future studies incorporating biochemical stimulation and multi-line validation will therefore be important.

## 5. Conclusions

In this study, we developed a human iPSC-derived SCO model that reproduces major features of HSs, including increased collagen deposition, disorganized ECM architecture, loss of hair follicle structures, and tissue contraction. SCOs were generated by inducing fibrotic remodeling in pre-established SKOs, resulting in a 3D human model of pathological scarring.

The model was designed based on the established roles of TGF-β1 and hypoxia in scar formation and was validated using histological, immunofluorescent, functional, and transcriptomic analyses. This iPSC-derived SCO system provides a robust and reproducible human 3D platform for investigating the mechanisms underlying pathological scarring and offers translational potential for evaluating therapeutic candidates and personalized antifibrotic drug screening.

## Data Availability

The original contributions presented in this study are included in the article/Supplementary Materials. Further inquiries can be directed to the corresponding authors.

## Supplementary Materials

The following supporting information can be downloaded at: https://www.mdpi.com/article/10.3390/cells15110969/s1, Supplementary Table S1: Differentially expressed genes between D130 SKOs and D130 SCOs.
